# Whole-Genome and Chromosome Evolution Associated with Host Adaptation and Speciation of the Wheat Pathogen *Mycosphaerella graminicola*

**DOI:** 10.1371/journal.pgen.1001189

**Published:** 2010-12-23

**Authors:** Eva H. Stukenbrock, Frank G. Jørgensen, Marcello Zala, Troels T. Hansen, Bruce A. McDonald, Mikkel H. Schierup

**Affiliations:** 1 Bioinformatics Research Center, Aarhus University, Aarhus, Denmark; 2 Plant Pathology Group, Institute of Integrative Biology, Swiss Federal Institute of Technology (ETH) Zurich, Zurich, Switzerland; Fred Hutchinson Cancer Research Center, United States of America

## Abstract

The fungus *Mycosphaerella graminicola* has been a pathogen of wheat since host domestication 10,000–12,000 years ago in the Fertile Crescent. The wheat-infecting lineage emerged from closely related *Mycosphaerella* pathogens infecting wild grasses. We use a comparative genomics approach to assess how the process of host specialization affected the genome structure of *M. graminicola* since divergence from the closest known progenitor species named *M. graminicola* S1. The genome of S1 was obtained by Illumina sequencing resulting in a 35 Mb draft genome sequence of 32X. Assembled contigs were aligned to the previously sequenced *M. graminicola* genome. The alignment covered >90% of the non-repetitive portion of the *M. graminicola* genome with an average divergence of 7%. The sequenced *M. graminicola* strain is known to harbor thirteen essential chromosomes plus eight dispensable chromosomes. We found evidence that structural rearrangements significantly affected the dispensable chromosomes while the essential chromosomes were syntenic. At the nucleotide level, the essential and dispensable chromosomes have evolved differently. The average synonymous substitution rate in dispensable chromosomes is considerably lower than in essential chromosomes, whereas the average non-synonymous substitution rate is three times higher. Differences in molecular evolution can be related to different transmission and recombination patterns, as well as to differences in effective population sizes of essential and dispensable chromosomes. In order to identify genes potentially involved in host specialization or speciation, we calculated ratios of synonymous and non-synonymous substitution rates in the >9,500 aligned protein coding genes. The genes are generally under strong purifying selection. We identified 43 candidate genes showing evidence of positive selection, one encoding a potential pathogen effector protein. We conclude that divergence of these pathogens was accompanied by structural rearrangements in the small dispensable chromosomes, while footprints of positive selection were present in only a small number of protein coding genes.

## Author Summary

The fungal wheat pathogen *Mycosphaerella graminicola* emerged in the Middle East 11,000 years ago, coinciding with host domestication. We sequenced the genome of the closest known endemic relative of *M. graminicola* infecting wild grass hosts. A comparative genome analysis allowed us to infer how speciation and host specialization processes have influenced pathogen evolution. The wild grass-adapted pathogen can infect wheat, but *M. graminicola* shows a significantly higher degree of host specificity and virulence in a detached leaf assay. The genomes of the pathogens are 7% divergent with a high degree of synteny in the 13 essential core chromosomes. However, structural rearrangements have strongly affected eight small dispensable chromosomes. These chromosomes also show altered rates of non-synonymous and synonymous substitutions. We found 43 genes showing evidence of positive selection. As the divergence of species occurred very recently, these genes are likely involved in host specialization or speciation. None of the genes have a known function, although one encodes a signal peptide and is a potential pathogen effector. We conclude that the genomic basis of the rapid emergence of the wheat-specialized pathogen *M. graminicola* has involved structural changes in the eight dispensable chromosomes and positive selection in a small number of genes.

## Introduction

We know little about the evolutionary processes leading to the origin and emergence of new fungal plant pathogens despite their significant impact on our food supply. Mass sequencing technologies that enable the acquisition of complete pathogen genome sequences provide a new approach to address questions related to the roles played by host specialization and speciation. Possible genetic changes associated with speciation in fungi include chromosomal rearrangements, duplications, gene loss and selection of particular genes [1]–[3]. By comparing the genomes of pathogens that have recently diverged and specialized onto different hosts we can quantify how much of the genome was affected by the process of divergence and identify genomic regions or particular genes that were involved in host specialization.

The wheat-infecting haploid fungus *Mycosphaerella graminicola* provides a unique model system for elucidating the processes underlying host specialization among different grass species and pathogen speciation on a relatively contemporary timescale. *M. graminicola* originated in the Fertile Crescent 10–11,000 years ago (90% highest posterior density interval 2,000 to 19,500 years ago) during the domestication of wheat and has coevolved and spread with its host globally [4]–[5]. The pathogen today causes one of the most important diseases of wheat. The emergence and “co-domestication” of *M. graminicola* was associated with an adaptation to wheat and an agricultural environment. Endemic descendants of the progenitor of *M. graminicola* are still found on wild grasses in the Middle East; however these “wild” pathogens show a broader host range than the “domesticated” wheat pathogen. The closest known relative of *M. graminicola* is named *M. graminicola* subspecies 1 (here abbreviated S1). S1 was isolated in Iran from the two grass species *Agropyron repens* and *Dactylis glomerata* growing in close proximity to fields planted to bread wheat (*Triticum aestivum*). Although *M. graminicola* is a frequent pathogen of wheat in Iran, no evidence of gene flow between S1 and *M. graminicola* was detected based on sequence analysis of six nuclear loci [5]. The first goal of the present study was to investigate the extent of genome divergence and patterns of chromosome evolution of S1 and *M. graminicola* that have occurred during the last 10–11,000 years.

The genome sequence of the Dutch isolate IPO323 of *M. graminicola* was recently completed [6]. The 40 Mb genome consists of 21 chromosomes that are completely sequenced. Karyotype analysis, crossing experiments and genetic analysis of different *M. graminicola* isolates showed that eight of the 21 nuclear chromosomes are dispensable in IPO323 [7], [8]. Dispensable chromosomes are found in other plant pathogenic fungi and have in some cases been shown to carry genes contributing to pathogenicity of isolates carrying the extra chromosomes [9]–[11]. It is not known how these small chromosomes originated in the genome of *M. graminicola* or whether they carry pathogenicity related genes. We anticipated that a comparison of the genomes of *M. graminicola* and S1 would provide new insight into the evolution of these dispensable chromosomes and allow us to make the first comparison of the genetic architecture and evolutionary patterns of essential and non-essential chromosomes in fungal pathogen genomes.

The second goal of our genome comparison was to identify genes that show evidence of positive selection. We hypothesize that genes directly involved in host-pathogen interactions, such as genes encoding effector molecules that may be involved in host specialization, will have diverged to a greater extent in response to selection by different host environments. We hypothesized that genes involved in speciation and reproductive isolation would also show evidence of selection. Earlier comparative genomics studies identified candidate loci affected by selection between fungal lineages, e.g. genes involved in sugar fermentation of domesticated and wild yeast strains [12], pathogenicity related genes of pathogenic and non-pathogenic Candida species [13] and genes involved in reproductive isolation between experimentally evolved Neurospora lineages [14].

We used paired end Illumina sequencing at 32X coverage to derive a high-quality assembly of the wild S1 species and aligned it to the *Mycosphaerella graminicola* genome to address the following questions: 1) Which structural genome changes have occurred since divergence and how can these be related to host specialization? 2) Have essential and dispensable chromosomes diverged to the same extent over the time course of 11,000 years? 3) Which genes have undergone rapid, adaptive evolution in S1 and *M. graminicola*? We confirm a recent and rapid development of reproductive isolation, report a strikingly different pattern of genome evolution for essential and dispensable chromosomes and identify genes and gene categories that appear particularly associated with host specialization or reproductive isolation.

## Results

### Test of host specialization on wheat

We used a detached leaf assay to measure pathogenicity of *M. graminicola* and S1 on wheat. The percentage of leaf area covered with pycnidia was used to quantify pathogenicity. The results demonstrate that both pathogens can infect wheat; however *M. graminicola* is considerably more pathogenic on wheat than S1. On average *M. graminicola* covered 50% of the infected wheat leaf area with pycnidia at 28 days after inoculation while S1 on average covered only 4% (Table S1).

### Assembly and alignment

A Velvet [15] de novo assembly of ∼45 million paired end Illumina reads resulted in a 32X average coverage of the S1 genome assembled into a 35 Mb draft genome sequence. The assembly consisted of 35,000 contigs with N50 = 78 K (longest contig 600 kb) (Table 1).

**Table 1:** Summary of S1 draft genome assembled using the Velvet assembler.

STIR04-3.11.1 (S1) assembly	
Assembly size	35 Mb
Coverage	32X
Assembled contigs	35,057
Contig N50 size	77,785 bp
Longest contig	589,124 bp

**Figure 1:**
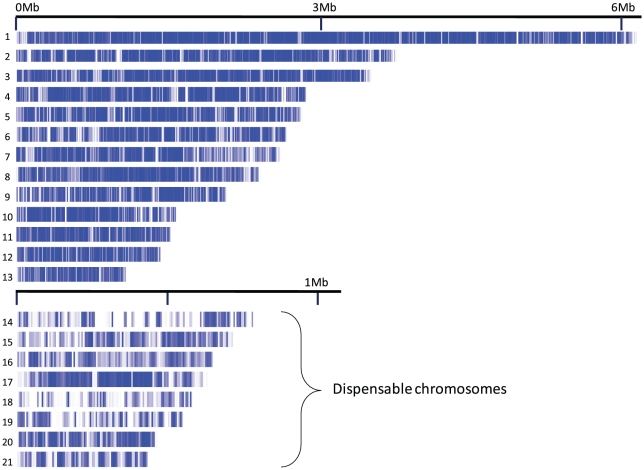
Nuclear chromosomes 1-21 of *Mycosphaerella graminicola*. Blue bars indicate regions aligned by contigs of the S1 de novo assembly.

We aligned 28 Mb of the S1 contigs to the 21 nuclear chromosomes of *M. graminicola* [6], representing 71% of the genome of *M. graminicola* and 80% of the S1 contigs (Figure 1). With paired end Illumina data an exhaustive analysis of genome synteny cannot be accomplished. Instead we investigated the microsynteny of each aligned contig and assessed the proportion of contigs that were entirely aligned to one reference sequence. If the total length of a contig can be aligned to one sequence in the reference it demonstrates synteny of the particular genome region. We found that 95% of the contigs >10,000 bp aligned to a syntenic region on a single chromosome, suggesting that synteny between the two species is extensive (Table 2, Table S2). However, contigs matching the dispensable chromosomes [8] more frequently exhibited breaks in synteny (25%) suggesting either more rearrangement on these chromosomes or higher rates of mis-assembly on either the reference genome or the Velvet assembled contigs. On chromosome 18 there was not a single S1 contig >1000 bp that could be aligned, indicating that this chromosome is absent in the S1 draft genome (data not shown).

**Table 2:** Chromosome-wide alignment.

Chromosome	Total bp	Total aligned	% of *M. graminicola* genome not aligned	% of non-repetitive DNA not aligned	Total genes	Aligned genes (% of total *M. graminicola* genes)	Aligned codons
Essential							
1	6119516	5099307	17	8	1981	1892 (96%)	793073
2	3877257	2927990	24	10	1136	1080 (95%)	460451
3	3521896	2782108	21	9	1071	975 (91%)	431247
4	2892740	2193557	24	10	821	728 (89%)	325481
5	2873973	2084833	27	11	778	688 (88%)	305799
6	2686295	1839353	32	12	692	631 (91%)	281065
7	2676027	1726677	35	25	766	577 (75%)	227916
8	2453492	1805622	26	9	689	622 (90%)	259319
9	2151231	1487710	31	13	604	520 (86%)	227199
10	1689705	1235803	27	15	516	459 (89%)	208847
11	1632476	1259536	23	14	488	422 (86%)	188990
12	1470463	1102283	25	12	408	359 (88%)	143622
13	1191065	831252	30	14	330	278 (84%)	106710
Dispensable							
14	774137	201550	74	59	114	25 (22%)	3909
15	641641	259126	60	39	86	42 (49%)	8679
16	609134	256062	58	46	88	50 (57%)	9567
17	586944	343425	41	20	78	44 (56%)	11393
18	574302	129688	77	64	64	-	-
19	551115	183800	67	57	87	29 (33%)	3790
20	474406	279705	41	24	79	49 (62%)	10699
21	410414	183792	55	36	58	39 (67%)	7784

Total length of *Mycosphaerella graminicola* chromosomes and total length of aligned sequence. Total number of annotated genes on *M. graminicola* chromosomes and genes aligned by the S1 sequence. Chromosomes were characterized as dispensable or non-dispensable (essential) as in [8].

Repeat masking identified a total of 18% repetitive sequence in the *M. graminicola* genome but only 4% in the S1 contigs (Table S3, Dataset S1). The *M. graminicola* genome may contain more repetitive DNA than the S1 draft genome, but it is also possible that the discrepancy in repeat content is due largely to difficulties in assembling and aligning larger repetitive regions. Based on the repeat masked sequence we calculated the quantity of non-repetitive DNA in *M. graminicola* to be 32 Mb compared to 33.6 Mb in S1, suggesting similar non-repetitive genome sizes for the two pathogens. The total genome size of S1 was estimated to be approximately 38.5 Mb based on pulsed field gel electrophoresis (see below). Assuming that non-assembled sequences comprise the repetitive fraction of the genome we find that S1 contains 4.8 Mb repetitive DNA, i.e. 12.5%, considerably less than in *M. graminicola*.

We found a strong correlation (Kendall's T = 0.56, p<2e-16) between repetitive DNA content and non-aligned DNA in *M. graminicola* on both essential and dispensable chromosomes (Figure S1). However, on the dispensable chromosomes large alignment gaps were also found in non-repetitive regions, suggesting that other factors such as deletions or insertions may underlie the higher extent of divergence on these chromosomes. Only 57% of the non-repetitive DNA on the dispensable chromosomes was aligned while 83.5% was aligned on the essential chromosomes (Table S2).

### Pulsed field gel electrophoresis

Electrophoretic karyotyping of S1 was carried out with pulsed field gel electrophoresis (PFGE). Chromosomes of different sizes (small: <1 Mb, medium size: 1–2.5 Mb and large: >2.5 Mb) were separated using different running conditions optimized for each size range. With the short run times we counted seven small chromosomes in the size range 0.6–0.9 Mb, nine medium-sized chromosomes between 1.5–2.3 Mb and four large chromosomes between 2.6–6 Mb. In total the PFGE analysis revealed 20 chromosomes resulting in a total genome size of approximately 38.4–38.6 Mb. This is similar to the genome size of 39 Mb of the sequenced IPO323 strain of *M. graminicola* [6]. Table 3 summarizes chromosomes numbers and sizes of S1 and in the reference *M. graminicola* reference isolate.

**Table 3:** PFGE of S1 isolate STIR04_3.11.1 and chromosomes in the reference genome of *M. graminicola* isolate IPO323.

Chromosome number	S1 3.11.1 Size (Mb)	IPO323 Size (Mb)
1	6	6.12
2	3.5	3.88
3	3	3.52
4	2.6	2.89
5	2.3	2.87
6	2.21	2.69
7	2.16	2.68
8	2.06	2.45
9	1.92	2.15
10	1.77	1.69
11	1.68	1.63
12	1.68	1.47
13	1.49	1.19
14	0.88	0.77
15	0.85	0.64
16	0.85	0.61
17	0.75	0.59
18	-	0.57
19	0.70	0.55
20	0.63	0.47
21	0.60	0.41
**Total genome size**	**37.6**	**39.86**

The S1 isolate contains only 20 chromosomes whereas the *M. graminicola* isolate has 21 chromosomes [6], [7].

According to our alignment and synteny analysis of the two genomes, the 13 largest chromosomes of S1 (1–6 Mb) are homologous to the 13 largest chromosomes of *M. graminicola*. Although homologous, the sizes of chromosomes 1–13 differ between the two genomes. The total size of the essential chromosomes in *M. graminicola* is 35.2 Mb and in S1 the total size of chromosomes >1 Mb is 32.4 Mb.

For the dispensable chromosomes our alignment suggests that these chromosomes are not homologous with the small chromosomes of S1 (<1 Mb). Indeed the separation of small chromosomes shows that S1 has 7 small chromosomes and that the size distribution of small chromosomes differs from the size of dispensable chromosomes in *M. graminicola*. The total chromosome length of small chromosomes in *M. graminicola* IPO323 is 4.6 Mb and for S1 it is 6.1–6.2 Mb.

In conclusion the karyotype analysis demonstrates similar overall genome sizes for S1 and *M. graminicola* and a homologous set of 13 large essential chromosomes. S1 has a large number of small chromosomes corresponding to the dispensable chromosomes in *M. graminicola*, although the poor alignment coverage indicates that the small homologous chromosomes may have undergone significant structural changes.

### Molecular evolution

Patterns of molecular evolution were described through estimates of substitution rates in the 28 Mb alignment including 9521 protein coding genes. Consistent with the structural differences between essential and dispensable chromosomes, we also found different patterns of molecular evolution between the two groups of chromosomes.

**Figure 2:**
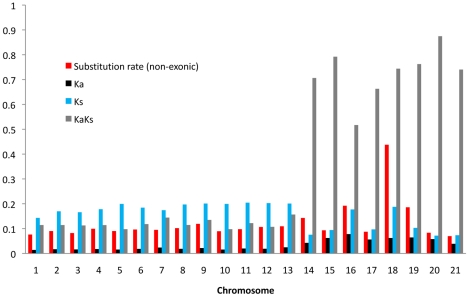
Average substitution rates in non-exonic sequences and average Ka/Ks ratio, Ka, and Ks for all aligned gene.

First, we calculated intergenic and synonymous substitution rates (Ks) in windows of 50 kb (chromosomes 1–8), 25 kb (chromosomes 9–13) and 10 kb (chromosomes 14–21) to infer patterns of neutral evolution along chromosomes (Figure S2). The overall genome divergence was 0.07 for the essential chromosomes and 0.087 for the dispensable chromosomes (Student's t-test, P<0.05). Also rates of non-synonymous substitutions Ka differed between essential and dispensable chromosomes, being significantly higher on the dispensable chromosomes (Figure 2). The high Ka rates on the dispensable chromosomes may be due to either enrichment in genes under positive selection or the presence of neutrally evolving pseudogenes. However, measures of Ks showed significantly higher values on the essential chromosomes compared to the dispensable chromosomes, indicative of other evolutionary forces influencing gene evolution.

Next, to estimate the frequency and distribution of putative pseudogenes and genes evolving under selection, we assessed Ka/Ks ratios for all genes under the assumption that genes with significant Ka > Ks are likely genes evolving under positive selection, genes with significant Ka < Ks are likely genes under purifying selection, while genes with Ka  =  Ks are likely to be pseudogenes. Under these assumptions, we observed a clear difference in the distribution of genes under positive and purifying selection as well as possible pseudogenes (Table 4). While the essential chromosomes showed a vast majority of genes (93%) under purifying selection and only a small number of genes (0.35%) with Ka > Ks, the dispensable chromosomes had a 10 times higher fraction of genes with Ka > Ks (4%) in addition to a higher fraction of putative pseudogenes (66%). The higher number of positively selected genes on the dispensable chromosomes may additionally represent potential pseudogenes. Experimental data will be needed to verify whether neutrally evolving genes are indeed pseudogenes. In summary, our data are consistent with a different pattern of molecular evolution on the eight dispensable chromosomes compared to the thirteen essential chromosomes, with different substitution rates in both intergenic and intragenic regions as well as an abundance of non-functional genes on the dispensable chromosomes.

**Table 4:** Ka/Ks ratios of aligned genes on essential and dispensable chromosomes.

	Total aligned genes	Non-synonymous substitutions	Synonymous substitutions	genes Ka>Ks 1)	freq. genes Ka>Ks	genes Ka<Ks	freq. genes Ka<Ks	genes Ka = Ks	freq. genes Ks = Ks
Essential chromosomes 1–13	9614	175546	508726	32	0.00374	8942	0.93	638	0.06636
Dispensable chromosomes 14–21	409	10876	5701	11	0.03912	118	0.29	274	0.66993

**1)** Z test of Ka > Ks, p<0.05.

### Determinants of substitution rates

**Figure 3:**
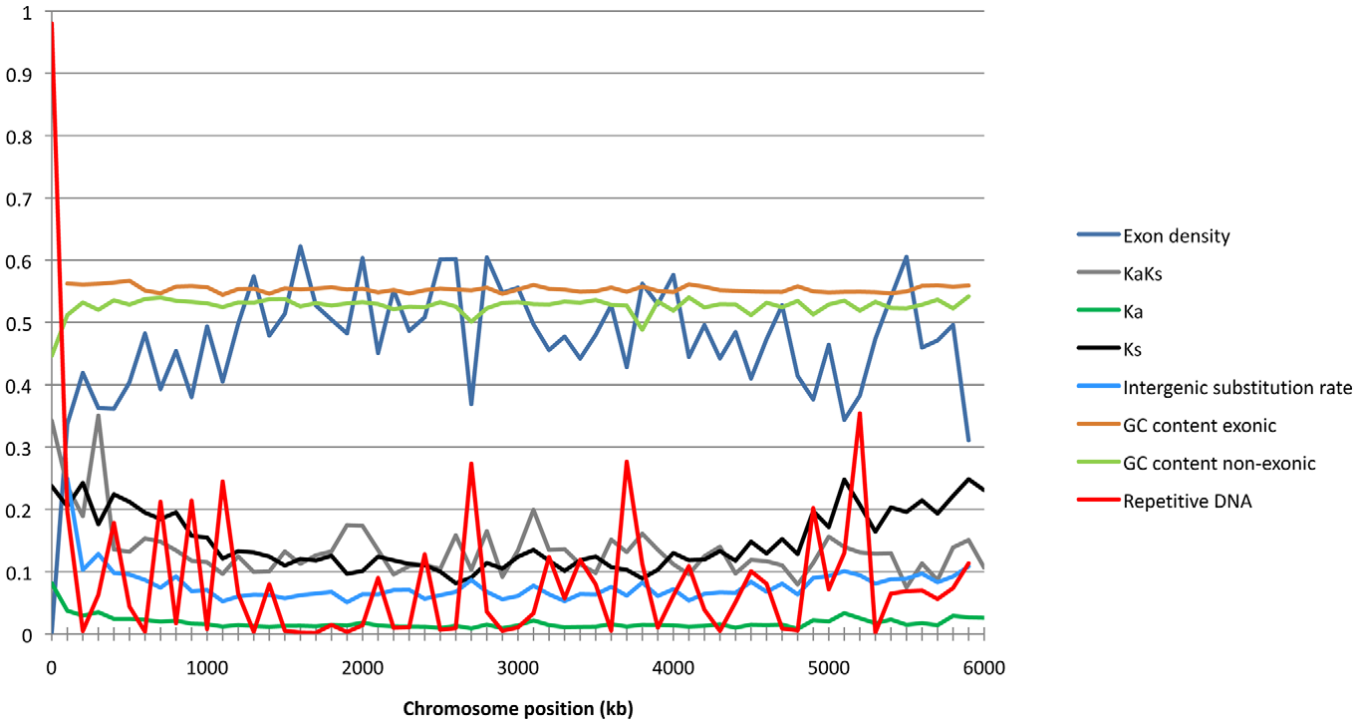
Sliding window of chromosome 1 showing exon density, Ka/Ks ratios, Ka, Ks, intergenic substitution rate, exonic and non-exonic GC-content, and frequencies of repetitive DNA as calculated in 100 K window sizes.

We applied the non-parametric test statistic Kendall's T and a multivariate test analysis to infer determinants of Ks, Ka and intergenic substitution rates including the distance from telomere, exon density, and GC content. Essential and dispensable chromosomes were analyzed as separate groups. We analyzed chromosome 1 separately to compare the overall chromosome pattern with that of the longest chromosome. Chromosomes 1 includes 17% of the DNA in essential chromosomes and 15% in the total chromosome set. For all parameters we found the same significant correlations on chromosome 1 as with the whole dataset of essential chromosomes (data not shown). The co-variation of genomic features in 100 K windows along chromosome 1 is illustrated in Figure 3.

**Figure 4:**
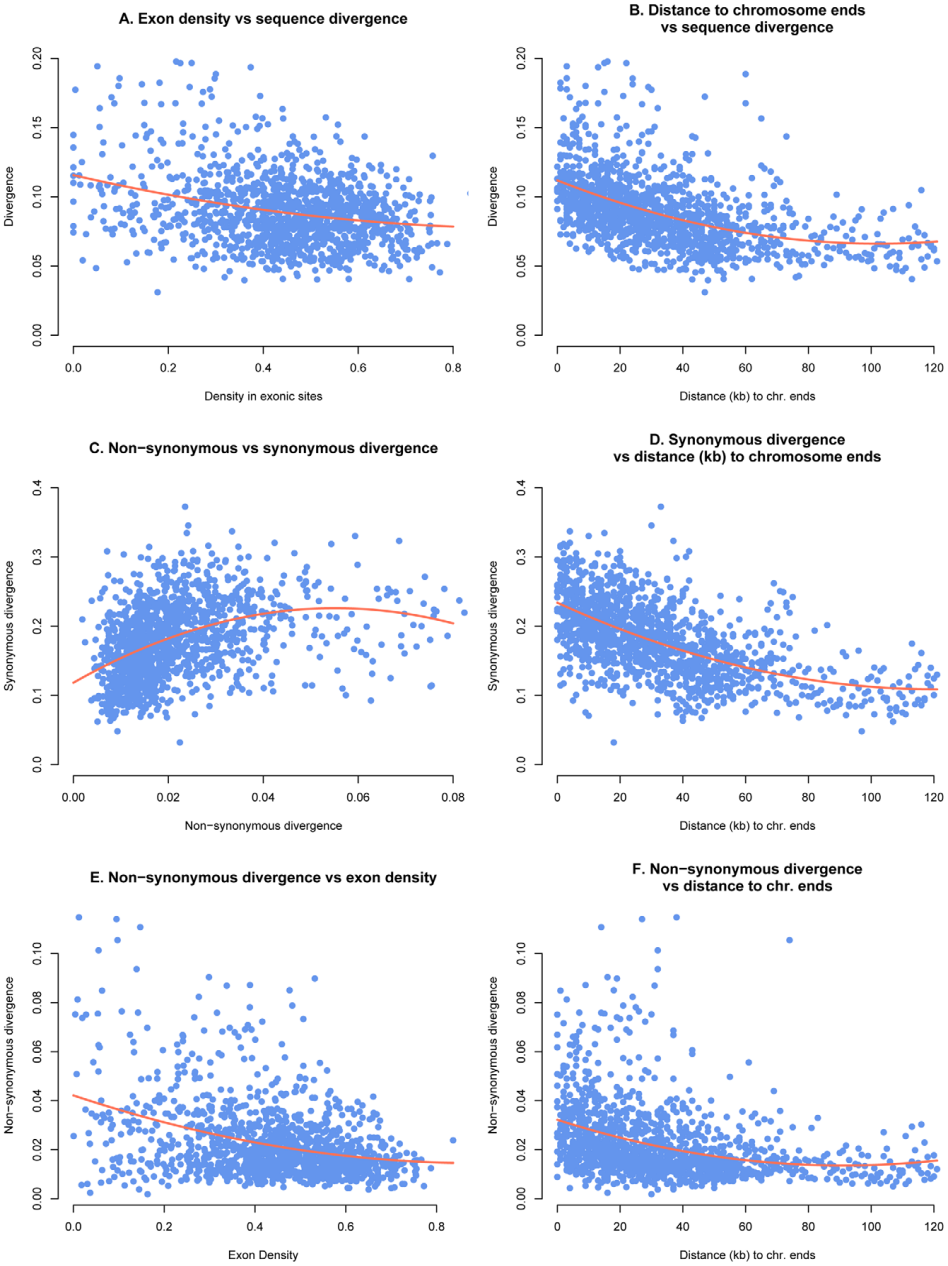
Genome parameters calculated in 25 kb windows and plotted with 2^nd^ order polynomial regression curve. A) Inter-genic substitution rate and exon density, B) inter-genic substitution rate and distance to telomeres, C) Ks and Ka, D) Ks and distance to telomeres, E) Ka and exon density, and F) Ka and distance to telomeres.

Ks as well as Ka were strongly positively correlated with intergenic substitution rates on both essential (T = 0.44, p<2.2e-16 and T = 0.35, p<2.2e-16 respectively) and dispensable (T = 0.39, p = 4.23e-10 and T = 0.32, p = 3.5e-07 respectively) chromosomes. The physical location of genes on the chromosomes is similarly correlated with evolutionary rates as we found strong negative correlations between both Ks and Ka and the distance to chromosomes ends (T = −0.2995481, p<2.2e-16 and T = −0.2995481, p<2e-16 respectively) on the essential chromosome (Figure 4). Similarly the intergenic substitution rate was negatively correlated with distance to chromosome ends on the essential chromosomes (T = −0.37, p<2.2e-16). Interestingly, while the distance to chromosomes ends explains a large part of the variation on essential chromosomes, there is no significant correlation with any of the substitution parameters on the dispensable chromosomes. This finding supports our hypothesis that the aligned sequences of dispensable chromosomes in S1 are not derived from chromosomes with colinear structure to the dispensable chromosomes of *M. graminicola*.

Exon dense regions had lower substitution rates including the intergenic substitution rate, Ka and Ks. We found significant negative correlations for these three parameters on the essential chromosomes (T = −0.2, p<2e-16; T = −0.2, p<2e-16 and T = −0.12, p = 1.85e-10 respectively). Ka/Ks ratios were also negatively correlated with exon density on the essential chromosomes (T = −0.2, p<2e-16 and T = −0.16, p<2e-16) implying that purifying selection is stronger in exon-dense regions. To the contrary, on the dispensable chromosomes exon density is not significantly correlated with Ks or Ka and only weakly negatively correlated with intergenic substitution rate (T = −0.12, p = 0.04).

The multivariate test also showed a markedly different pattern of parameter associations on the essential versus dispensable chromosomes. For Ks there was a significant correlation of Ka (p<2e-16), intergenic substitution rate (p = 2.21e-08), GC content (p = 0.001), exon density (p = 0.001) and the distance to chromosome ends (p<2e-16). However, on the dispensable chromosomes only Ka (p = 4.71e-12) and intergenic substitution rates (p = 1.02e-07) were significantly correlated with Ks. The intergenic substitution rate on the essential chromosomes was determined by the same parameters as Ks except that there was no association with exon density (Ka: p = 4.48e-06, Ks: p = 2.21e-08, GC content: p = 7.48e-15, distance to chromosome ends: p = 0.0129). On the dispensable chromosomes only GC content (p = 3.47e-07) and Ks (p = 1.02e-07) was correlated with intergenic substitution rate.

### Gene evolution

We compared the distribution among 26 functional EuKaryotic Orthologous Groups (KOG classes, The Joint Genome Institute) of annotated genes in *M. graminicola* and of the homologous genes in S1 to test whether some gene classes were either enriched or reduced in the number of aligned genes (Table S4). The null hypothesis was that the distribution of genes in KOG classes would not differ between aligned and non-aligned genes due to randomness in the divergence and gain/loss of genes. We found a statistically significant difference in the distribution of genes among KOG classes (p<0.00002, *X* ^2^ = 65.74, d.f. 25). In particular the number of aligned genes encoding proteins classified as extracellular structures and proteins involved in inorganic ion transport and metabolism were lower than expected, suggesting that these groups of genes are more diverged between S1 and *M. graminicola*, possibly including the loss or gain of genes. In contrast, the number of genes encoding proteins with unknown function and proteins with signal peptides were highly conserved in numbers of annotated and aligned genes.

A goal of our genome comparison was to identify candidate genes involved in host specialization and to search for evidence of reproductive isolation between *M. graminicola* and S1. We hypothesized that genes involved in host specialization evolved under positive selection in the two pathogens as a consequence of selection for adaptation to different host species. We therefore focused our search on genes with Ka > Ks as candidate genes that have undergone positive selection. 43 genes showed significant evidence of selection using a Z test (p<0.05) (Table S5). Of these 43 genes, 32 were located on the essential chromosomes and 11 were on dispensable chromosomes. On the essential chromosomes 26 genes had EST support compared to only two genes on the dispensable chromosomes. None of the 43 genes were grouped in the KOG classes or described by a known gene function, suggesting that these positively selected genes are unique to the *Mycosphaerella* grass pathogens.

### Candidate effectors

To search for potential effector genes we used the SignalP software [16] to assign signal peptide prediction scores and putative cleavage sites to amino acid sequences of *M. graminicola* and S1. We identified 1217 proteins with a predicted signal peptide among the annotated genes in *M. graminicola*. Of these 802 had a Hidden Markov Model probability score > = 0.9. Of the 802 proteins with the highest scoring signal peptides, 787 were shared between S1 and *M. graminicola.*

We assessed the distribution of genes encoding signal peptides on essential and dispensable chromosomes and found that these genes were more likely to be located on the essential chromosomes (770 with signal peptides) compared to the dispensable chromosomes (17 with signal peptides) (Table 5).

**Table 5:** The number and percentage of genes on essential and dispensable chromosomes encoding signal peptides and non-signal peptides and the average Ka and Ks values for the two groups of genes on essential and dispensable chromosomes.

	signal peptide (HMM>0.9)				Non signal peptide			
	No of genes	Ka	Ks	Ka/Ks	No of genes	Ka	Ks	Ka/Ks
Essential chromosomes	770	0.026	0.188	0.168	9510	0.0216	0.184	0.138
	7.5%				92.5%			
Dispensable chromosomes	17	0.036	0.048	0.378	637	0.057	0.102	0.741
	2.5%				97.5%			

We hypothesized that adaptation to the different hosts of *M. graminicola* and S1 would lead to differences in secreted proteins, including effector molecules that interact directly with their respective host molecules. We therefore estimated rates of Ka and Ks as well as the overall ratio of Ka/Ks in the 787 shared genes encoding signal peptides and next analyzed the distribution of genes with Ka>Ks among these 787 shared genes (Table 5). One gene on chromosome 3 with a Ka > Ks (Z- test, p<0.05) was predicted to encode a secreted protein with 76 amino acids. Based on its small size, the number of cysteine residues and the Ka:Ks ratio, we consider this gene a likely candidate for encoding an effector protein that is differently recognized in *A. repens* and *T. aestivum*.

### Mating type locus

Host adaptation has played an essential role in the speciation of *M. graminicola*. However as the divergence of *M. graminicola* and S1 occurred between sympatric populations [5] we hypothesized that selection for reproductive isolation has similarly played an important role in the split of populations. We focused on the mating type locus of *M. graminicola* [17] and S1 as a locus that could have been affected by selection for reproductive isolation. Both strains had the MAT1-1 mating type. The S1 mating type locus was submitted to Genbank (accession number GU263885). We analyzed a 7 kb region on chromosome 13 spanning the *MAT1-1* locus and assessed the number of polymorphic sites. The overall nucleotide divergence in the intergenic DNA was 0.056, lower than the overall intergenic genome divergence of 0.09. In the protein coding *MAT1-1* gene we identified a total of twelve mutations between *M. graminicola* and S1, five non-synonymous and seven synonymous, resulting in a Ka/Ks ratio of 0.23 and a nucleotide diversity of 0.001 (Table S6). The low level of diversity in the mating type gene suggests that the reproductive isolation between the two lineages has not affected the nucleotide sequence of the *MAT1-1* gene. However, our recent investigation of the other idiomorph *MAT1-2* in *M. graminicola* and S1 revealed a surprisingly high number of amino acid changes between the two species, indicating that between species mating incompatibility may be mediated through differences in the *MAT1-2* gene (Stukenbrock et al, in prep).

To differentiate between inter and intra-specific diversity in the mating type locus we assessed the number of polymorphisms and substitutions in population samples of *M. graminicola* and S1. For the population sampling we included both a 1005 bp non-coding locus flanking the *MAT1-1* gene as well as 511 bp of coding DNA in the *MAT1-1* gene. In general the intra-specific diversity in both species is very low. In the protein coding sequence we detected only two mutations (one synonymous and one non-synonymous) within the *M. graminicola* population samples and none in the S1 population. In the 1005 bp non-coding region there were 4 polymorphisms in the *M. graminicola* population sample and none in S1. Between species we detected 12 substitutions in the coding sequence and 23 substitutions in the 1005 bp non-coding region. There were no intra-specific polymorphisms shared between S1 and *M. graminicola* in the sequenced regions.

## Discussion

The genome comparison conducted in this study suggests that the recent emergence of *M. graminicola* as a specialized wheat pathogen was associated with genomic changes involving mainly DNA located on the smallest chromosomes (<1 Mb). The remainder of the genome has maintained a high degree of identity between *M. graminicola* and S1. This genome comparison included only two fungal individuals. It is well known that *M. graminicola* exhibits not only intra-specific presence/absence polymorphism in the dispensable chromosomes, but also size polymorphisms [8], [18]. The lack of synteny and the different size distribution of the smallest chromosomes in S1 suggest that this set of chromosomes is not entirely homologous to the small chromosomes in *M. graminicola*. The small chromosomes instead show a fragmented pattern of aligned sequences that have evolved in a different manner compared to the essential chromosomes. So far we do not know whether the seven small chromosomes in S1 are dispensable, but PFGE analyses have revealed size polymorphisms among the small chromosomes in other S1 isolates. More intra-specific data will be needed from both species to better understand the karyotype-level structural changes that occurred during speciation.

Our data suggests that the structural changes between S1 and *M. graminicola* have exceeded the molecular evolution at the nucleotide level. It was previously reported that rates of structural and molecular evolution are correlated in vertebrates, nematodes and arthropods [19]–[21]. However, the present study as well as previous genome comparisons [3], [22], [23] illustrate that rates of structural evolution can be accelerated relative to rates of molecular evolution in fungi.

The speciation of *M. graminicola* and S1 occurred 10–11,000 years ago representing approximately 10,000 pathogen generations; however this significant differentiation of genomes may have occurred over a longer period of time or as a consequence of host domestication and a very rapid split between pathogen lineages as *M. graminicola* co-evolved with wheat to become host-specialized. Our pathogenicity tests show that S1 causes less disease and forms many fewer reproductive structures on wheat, demonstrating that specialized adaptation to wheat is a trait that emerged in *M. graminicola* after the split between the two pathogen species. This host specialization may have been a driving factor in the process of speciation. The average genome divergence of 7% is consistent with a speciation time of 11,000 years as previously estimated using the Isolation with Migration (IM) coalescence model [5], [24]. The divergence time is the speciation time plus average time to coalescence in the common ancestral species [25]. The mean coalescence time in the ancestral species is 2N generations, where N is the effective population size of the ancestral species. Assuming an effective population size in the ancestral species of 15,000 (Stukenbrock et al, unpubl) we find that the average divergence time is 11,000 years plus 2 * 15,000 = 41,000 years (assuming on average one generation per year). This would correspond to an average substitution rate of 0.07/82,000 = 8.5×10^−7^ nucleotide substitutions per generation consistent with experimentally measured mutation rates in yeast [26].

A striking finding of our genome comparison is the highly different patterns of nucleotide evolution on the essential and dispensable chromosomes. The dispensable chromosomes share several features characteristic of B-chromosomes in plants, including more structural changes and an accumulation of repetitive DNA and pseudogenes [27]. Previous studies have reported an irregular meiotic behavior of this group of chromosomes as well as a different recombination pattern caused by the absence of pairings between homologous chromosomes in crosses involving isolates with different chromosome combinations [8]. We believe that the different evolutionary patterns of essential and dispensable chromosomes observed here are due to the irregular transmission of the dispensable chromosomes. Chromosomes that exist in the pathogen population at lower frequencies will as a consequence have a lower effective population size compared to the essential chromosomes that are always present in all individuals. If the small chromosomes were also dispensable in the common ancestor of *M. graminicola* and S1, they would similarly have had a lower effective population size. In the modern species this would be observed as less average divergence, which is indeed the pattern we observe when comparing essential and dispensable chromosomes. Distinct evolutionary patterns of essential and dispensable chromosomes have also been demonstrated for another fungal plant pathogen. In a comparison of *Fusarium* genomes it was also shown that dispensable chromosomes have different sequence characteristics including a higher content of unique genes and a different codon usage compared to core chromosomes of the pathogens [11]. The authors suggested that horizontal transfer of chromosomes between *Fusarium* species led to the emergence of new host specific lineages by the acquisition of new pathogenicity related genes. Genome plasticity of *M. graminicola* and closely related species may similarly play a role in the evolution of different host specificities and horizontal gene transfer from distantly related species has been proposed as an origin for the dispensable chromosomes [6]. Sequencing of additional isolates and species will enable us to further investigate the origin and evolution of the dispensable chromosomes in *Mycosphaerella*.

On the essential chromosomes we find significantly higher Ks compared to intergenic substitution rates. The intergenic chromosome regions were on average only 70% as diverged as synonymous sites in exons. The fact that our alignment did not include rapidly evolving repetitive DNA may affect our average estimates of intergenic substitution rates. However, our findings suggest that a large fraction of the intergenic DNA on the essential chromosomes evolves under selective constraints. The importance of natural selection on genes also manifests itself as a lower synonymous rate in gene dense regions, suggesting a combination of background selection and selective sweeps. On the contrary, Ks on the dispensable chromosomes was significantly lower than Ks on the essential chromosomes. This does not necessarily imply a lower mutation rate on the dispensable chromosomes but, as mentioned above, is likely a consequence of a smaller effective population size for the small chromosomes. In the ancestral species of *M. graminicola* and S1 this would result in less polymorphism on the dispensable chromosomes compared to the essential chromosomes and thus also less average divergence. In the dispensable chromosomes we additionally find that Ks and the intergenic substitution rate were of the same size, most likely reflecting the neutral evolution of the large number of pseudogenes.

Our analysis of gene evolution shows that ∼70% of genes on the dispensable chromosomes are putative pseudogenes. Because the present study does not include an out-group, we are not able to say if the genes are evolving to become pseudogenes only in *M. graminicola* while maintaining function in S1. Future studies will elucidate the role of these genes in S1. Among the 11 genes on the dispensable chromosomes that show evidence of positive selection, only two were supported by EST data. This distribution of non-functional genes strongly supports the hypothesis that a large fraction of the DNA present on the eight small chromosomes is redundant i.e. not playing an essential role in fitness of the pathogen. However the persistence of these chromosomes in the genomes of the pathogens indicates that some of the genes on these chromosomes may still play an important role for the pathogen species.

We found that measures of genome variation including intergenic substitution rates, Ks and Ka were unevenly distributed along chromosomes, with sequence divergence increasing towards the chromosome ends. Similar patterns were found in yeast using whole genome oligonucleotide arrays including both coding and non-coding DNA [28]. Genes with increased Ka/Ks ratios were also located closer to chromosome ends compared to more conserved genes, suggesting that the chromosomal location of a gene can influence its evolutionary potential.

While the infection biology of S1 has not yet been studied intensively, we believe *M. graminicola* and S1 are likely to share many features in their disease cycles because they are so closely related, differing mainly in their specific interactions with particular host molecules. Extensive gene loss has been reported in other comparative genome studies of fungi [3], [29] and we hypothesize that gene loss also played a significant role in the genome divergence of S1 and *M. graminicola*. 10% of the genes annotated in *M. graminicola* were not aligned by S1 contigs and we likewise could not align all assembled S1 contigs to the *M. graminicola* genome, demonstrating the presence of similar S1 specific sequences. We identified 43 genes with a signature of positive selection. Our stringent significance thresholds for Ka>Ks could lead us to miss genes with less dramatic signatures of positive selection. For example our approach to test Ka>Ks may not be sensitive enough to detect positive selection that affects only a few codons in a gene. The set of genes we identified in this analysis have significantly diverged between *M. graminicola* and S1. None of these 43 positively selected genes has a known protein function. Only one of these genes encodes a secreted protein and has the characteristics expected for a possible effector molecule. The pooled set of signal peptides has an increased ratio of KaKs demonstrating an increased evolutionary rate compared to the remaining fraction of protein coding genes. Interestingly the vast majority of signal peptides are located on the essential chromosomes, suggesting an essential function for the proteins encoded by these genes.

The process of speciation between S1 and *M. graminicola* included the evolution of reproductive barriers between the two pathogens. The intra and interspecific comparisons reported here and in our previous study of S1 and *M. graminicola* provide no evidence for contemporary genetic exchange between the pathogens [5]. Though many genes are known to be involved in ascomycete mating, we focused on the gene encoding the mating type protein MAT1-1. The mating type gene is located in a chromosomal region of lower diversity compared to the general chromosome divergence. Low levels of diversity at the mating type loci and flanking regions have been reported in other fungi as a consequence of less recombination at the MAT locus in heterothallic fungi [30], [31]. While the *MAT1-1* locus appears to be conserved across species borders, our ongoing characterization of the *MAT1-2* gene in the two species suggests that reproductive barriers can be mediated at least partially through a high number of amino acid changes in the MAT1-2 protein (Stukenbrock et al, in prep).

This is the first comparative genome analysis of plant pathogens that have recently diverged in response to host domestication. Although we found a high degree of nucleotide similarity between the two genomes, our findings suggest that the divergence of these pathogens has been accompanied by structural rearrangements in their genomes and strong positive selection on a small set of genes with unknown function. Ongoing resequencing of additional S1 and *M. graminicola* strains will allow us to more precisely differentiate intraspecific versus interspecific diversity at the genome level. Genome sequencing of “S2”, another *Mycosphaerella* species infecting wild grasses in the Middle East, will provide additional opportunities to understand the origin of dispensable chromosomes as well as to determine how “natural” speciation between S1 and S2 affected genome structure compared to the “human mediated” speciation that led to *M. graminicola*.

## Materials and Methods

### Pathogenicity assays

Pathogenicity assays were carried out using detached leaves [32]. An Iranian *M. graminicola* isolate (STIR04-A26b) and the sequenced Iranian S1 isolate described in this paper (STIR04-3.11.1) were inoculated onto the highly susceptible winter wheat cultivar Drifter using a spore concentration of 10^7^ per mL. The percentage of leaf area covered by pycnidia at 28 days after inoculation was used as a measure of pathogenicity. The assay was repeated three times.

### Illumina paired end sequencing and assembly

DNA from the S1 isolate STIR04-3.11.1 collected from *Agropyron repens* in Ardabil, Iran in 2004 was used for Illumina sequencing. The fungal draft genome was sequenced by Beijing Genomics using the Illumina genome analyzer system. 10 ug DNA was used for the construction of two paired end libraries. Read lengths of the two libraries were 35 and 44 base pairs with average insert sizes of 200 and 400 base pairs respectively. A total of ∼45 million paired end Illumina reads (10,909,460 reads of 35 bp with insert size 200 bp and 34,784,978 reads of 44 bp with insert size 500 for a total of 1.19 Gb DNA sequence) were collected for assembly.

For computation of the de novo assembly we applied the Velvet 0.6 assembler [15]. Expected insert lengths were between 71–700 bp for 35-mers and 90–900 bp for 44-mers, expected short read k-mer coverage was set to 20 and the k-mer length 21. For quality filtering of the assembly we calculated a weighted average of the k-mer coverage of all assembled contigs. The distribution of k-mer coverage was then plotted to determine a cut off value for low quality contigs. We set the cut off value at 25X k-mer coverage and removed all contigs with an average coverage below this value (Figure S3). Quality filtering removed smaller contigs (0.003%) of low coverage.

### Alignment of the de novo S1 assembly to the *M. graminicola* genome sequence

The de novo S1 assembly was aligned to the genome sequence of the *M. graminicola* isolate IPO323 [6] using the Blastz software package [33]. We used the finished version of the *M. graminicola* genome (v2.0) containing 21 chromosomes, 20 of which are represented telomere to telomere with 5 gaps and a total genome size of 39.7 Mbp. For the pair wise alignment of the de novo assembled S1 contigs and the *M. graminicola* genome we used the program LASTZ_D. LASTZ_D is an extended version of the genome alignment software BLASTZ that performs alignment of genome sequences as well as contigs of varying lengths (http://www.bx.psu.edu/miller_lab/dist/README.lastz-1.01.50). Different parameter settings were tested to determine optimal parameters for the alignment of S1 and *M. graminicola*. The best alignment was obtained using the yasra85 option (seeding option T = 2; match and mismatch score —match = 1,2; gap costs O = 4 and E = 1; threshold for gapped extension Y = 20; score threshold for high scoring segment pairs (HSPs); gap threshold for gapped extension L = 30; expected identity —identity = 85).

The LASTZ_D aligner creates a list of pairwise alignments in maf format. We used the program Single_Cov2 incorporated in the software package TBA to identify overlapping regions in maf files and to generate concatenated chromosome-wide maf alignments [34]. Finally, the program multiz-tba from the TBA program package was used to construct fasta alignments of each *M. graminicola* chromosome.

We extracted S1 unique sequences not aligned to the *M. graminicola* genome by comparing the original de novo assembled contigs and the maf alignment files. A search for homologous nucleotide sequences in the S1 unique sequences was performed by the NCBI netblast software BLASTN (http://www.ncbi.nlm.nih.gov/blast/docs/netblast.html#5.3.2). The program searches a nucleotide database for homologous matches of input query nucleotides, in this case the not aligned S1 contigs. The NCBI database of fungal nucleotide sequences and a *M. graminicola* EST library [35] was used as target for the BLASTN search.

### Synteny

We applied two approaches to measure synteny in chromosome alignments. Initially we used the MUMMER software package to compute the degree of synteny between the draft genome sequence of S1 and the *M. graminicola* genome (Delcher et al, 2002). The NUCmer pipeline was applied to generate summary information about coordinates of all S1 contigs in the alignment. We counted the number of contigs >1000 bp aligned to one, two or ≥ three chromosomes and determined the distribution of broken and unbroken contigs on essential and dispensable chromosomes (Table S1).

Additionally, we used coordinates of contigs and *M. graminicola* chromosomes from maf alignment files to generate an alignment profile for each contig indicating whether a given contig was aligned in its full length or aligned in fragments on one or more chromosomes.

### Pulsed field gel electrophoresis

A modified non-protoplast protocol for generating karyotypes of fungi was used to generate an electrophoretic karyotype of S1 [36]. DNA plugs were prepared by embedding intact spores in agarose and treating with EDTA, SDS and protease XIV (Sigma, Switzerland) at 60°C for 48 h. PFGE was conducted with a contour-clamped homogeneous electric field (CHEF) Biorad DR-II apparatus in 120 ml 1X TAE, 0.8 to 1% Agarose (Invitrogen, Switzerland) with the following running conditions: for small chromosomes <1 Mb, Temp 13° to 14°C, 180 V with a ramped 60–120 sec switching interval for 27 to 28 h; for medium-sized chromosomes 1–2.5 Mb, Temp 3° to 14°C, 90 to 100 V with 500 s switching interval for 50 to 76 h; for large chromosomes >2.5 Mb, Temp 14°C, 70 V with 20–30 min interval switching for 48 to 52 h. Standard chromosome size markers used were from *Saccharomyces cerevisiae* (small chromosomes), *Hansenula wingei* (medium chromosomes) and *Schizosaccharomyces pombe* (large chromosomes). We repeated the electrophoresis three times for small and medium-sized chromosomes and two times for the large chromosomes with slightly altered running conditions for optimal chromosome separation. Gels were stained with ethidium bromide for 20 min. and karyotypes were made visible using a UV transilluminator. Chromosome bands were identified with visual inspection and gel pictures were further analyzed using the software QuantityOne (Biorad laboratories).

### Identification of repetitive DNA

We used the software RepeatScout for de novo identification of repeat families in the fungal genome sequences [37]. The RepeatScout algorithm is based on a pairwise search of repeated sequences. The program constructs a library of repetitive families distinguishing between low complexity repeat families, tandem repeat and multicopy exon families, segmental duplications and transposon families. Only repeat families with more than 10 copies are maintained while very short repetitive sequences are discarded. The resulting repeat library generated from the reference sequence of *M. graminicola* was used with the RepeatMasker software (A.F.A. Smit, R. Hubley and P. Green; RepeatMasker at http://repeatmasker.org) to mask repetitive sequences in both the *M. graminicola* genome and the S1 draft genome (including both the aligned and the non-aligned sequences).

### Calculation of intergenic, synonymous, and non-synonymous substitution rates

Estimates of divergence in non-exonic DNA were calculated as the percentage of diverging sites in the genome alignment. For analysis of coding sequences we used annotation information from the *M. graminicola* v2.0 genome. The genome contains 10,952 predicted genes functionally annotated through the Joint Genome Institute annotation pipeline. We estimated synonymous (Ks) and non-synonymous (Ka) mutation rates in fully aligned genes with no internal stop codons. We used the Nei and Gojobori method [38] as well as the Yang and Nielsen method [39] as implemented in PAML [40] to calculate Ka and Ks. The major difference between the two estimates is that the Yang and Nielsen method accounts for transition/transversion bias and codon usage bias. In order to group genes into conserved (Ka < Ks), positively selected (Ka > Ks) and putatively non-functional pseudogenes (Ka  =  Ks) we assessed the ratio of Ka/Ks for all genes and used a two tailed Z-test and Fisher's Exact test to compute a p value for each estimate. Information about EST support of each candidate gene was obtained from the Joint Genome Institute's annotation of the reference genome.

### Development of genome browser and regression analysis of genome parameters

We developed a genome browser to further analyze the genome alignment. In the genome browser we implemented calculations of substitution rates, GC content, exon density and frequencies of repetitive DNA and alignment gaps. The different parameters can be calculated and visualized in different window sizes, providing an important tool to extract and test data from the chromosome alignment. Next we applied the non-parametric Kendall's rank correlation to estimate the strength of dependence between genome variables and finally a multivariate analysis to estimate the effect of multiple variables on substitution rates in coding and non-coding regions. Estimates of exon density, Ka, Ks, Ka/Ks, inter and intragenic GC content, inter and intragenic substitution rates, and frequencies of repetitive DNA and alignment gaps were calculated in 100 K, 50 K and 25 K windows. Estimates were compared for all three window sizes to determine consistency at different scales.

### Signal peptides

Signal peptides and cleavage sites were predicted in 11,400 *M. graminicola* proteins by both the hidden Markov Model (HMM) and the neural network algorithms of the SignalP software [16], [41]. Predicted signal peptides with a HMM probability > = 0.9 were divided into shared and not-shared proteins according to the alignment between S1 and *M. graminicola*. We assessed the frequency of cysteine residues in proteins with <150 amino acids and with predicted signal peptides in order to identify putative shared effectors of S1 and *M. graminicola*. Ka/Ks ratios were estimated for all genes encoding signal peptides.

### MAT1-1 analysis

To test whether divergence of S1 and *M. graminicola* was associated with changes at the mating type locus we analyzed sequence divergence in a 7500 bp aligned fragment encompassing the mating type gene. Both the sequenced *M. graminicola* isolate IPO323 and the sequenced S1 isolate have the MAT1-1 idiomorph. The *MAT1-1* gene is 2839 bp in size and contains a single open reading frame encoding a putative α1-domain protein of 297 amino acids [17]. The program DnaSP v.5 was used to estimate nucleotide differences between the two sequences in both the coding and non-coding regions [42].

To estimate the level of intra-specific diversity we amplified and sequenced two loci flanking the mating type gene from 116 *M. graminicola* isolates and 22 S1 isolates. Primers, PCR conditions and sequencing methods were as previously described [43], [44]. Intra and intergenic diversity of the *MAT1-1* gene was assessed in DNAsp.

## Supporting Information

Dataset S1 Library of repetitive sequences in S1 generated by RepeatScout.
(0.30 MB TXT)

Figure S1 Regression analyses of chromosome parameters: Frequency of repetitive sequences (repetitions) and distance to chromosome ends (dist); frequency of repetitive sequences and exon density; frequency of repetitive sequences and GC content; frequency of repetitive sequences and alignment gaps (N-content); alignment gaps and GC content; alignment gaps and exon density.
(0.30 MB TIF)

Figure S2 Synonymous (a) and intergenic (b) substitution rates across aligned chromosomes.
(0.11 MB PDF)

Figure S3 Average coverage of de novo assembled S1 contigs. Distribution of Velvet contig coverage.
(0.03 MB PDF)

Table S1 Pathogenicity test of *M. graminicola* (STIR04-A26b) and S1 (STIR04-3.11.1) on wheat. The experiment was carried out with three repetitions where the percentage of leaf area covered by pycnidia was assessed.
(0.01 MB PDF)

Table S2 Measures of synteny by counts of S1 contigs that align to one chromosome, two chromosomes, or three or more chromosomes. Counts are further divided into contigs aligning to essential chromosomes, dispensable or both types of chromosomes.
(0.05 MB PDF)

Table S3 The fraction of repetitive and non-repetitive DNA that is aligned in the S1-M. graminicola genome alignment.
(0.01 MB PDF)

Table S4 GO classification of annotated genes in the Mycosphaerella graminicola-S1 alignment (EuKaryotic Orthologous Groups http://genome.jgi-psf.org/Mycgr3/Mycgr3.home.html).
(0.03 MB PDF)

Table S5 The 43 genes identified to have significant Ka > Ks in the M. graminicola and S1 alignment.
(0.07 MB PDF)

Table S6 Detailed sequencing and analysis of the MAT1-1 locus.
(0.01 MB PDF)
