# Supplementary figures and images for: Whole-Genome and Chromosome Evolution Associated with Host Adaptation and Speciation of the Wheat Pathogen Mycosphaerella graminicola
Source: PLoS Genet. 2010 Dec 23;6(12):e1001189. doi: 10.1371/journal.pgen.1001189 (PMC3009667; doi:10.1371/journal.pgen.1001189)

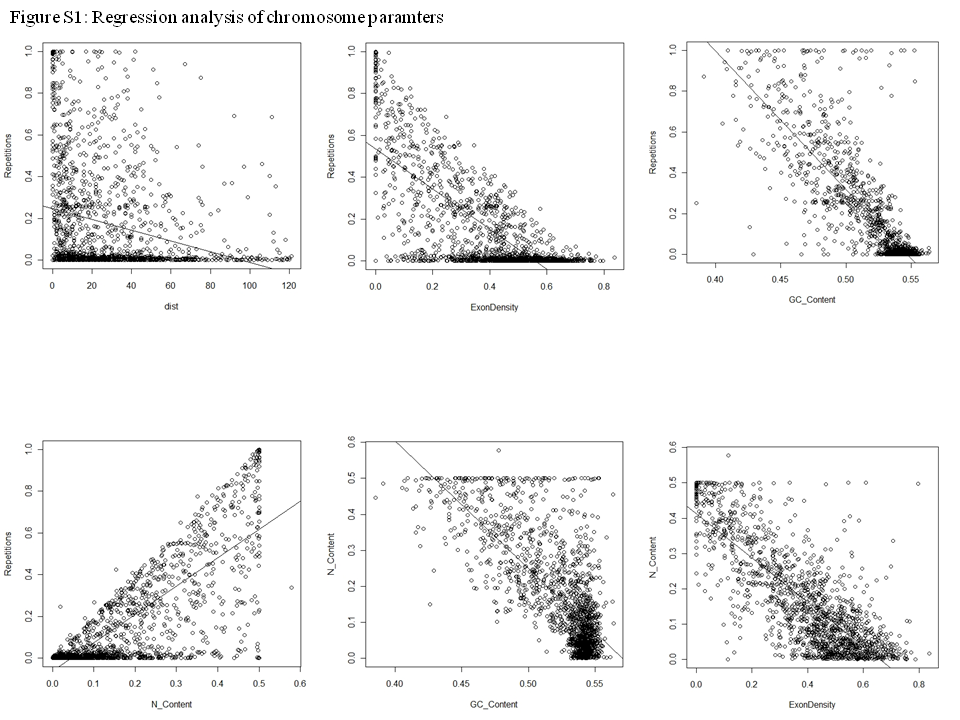

Supplement: Figure S1 — Regression analyses of chromosome parameters: Frequency of repetitive sequences (repetitions) and distance to chromosome ends (dist); frequency of repetitive sequences and exon density; frequency of repetitive sequences and GC content; frequency of repetitive sequences and alignment gaps (N-content); alignment gaps and GC content; alignment gaps and exon density. (0.30 MB TIF) [file pgen.1001189.s002.tif]

# Weighted average of coverage on contigs of different lengths

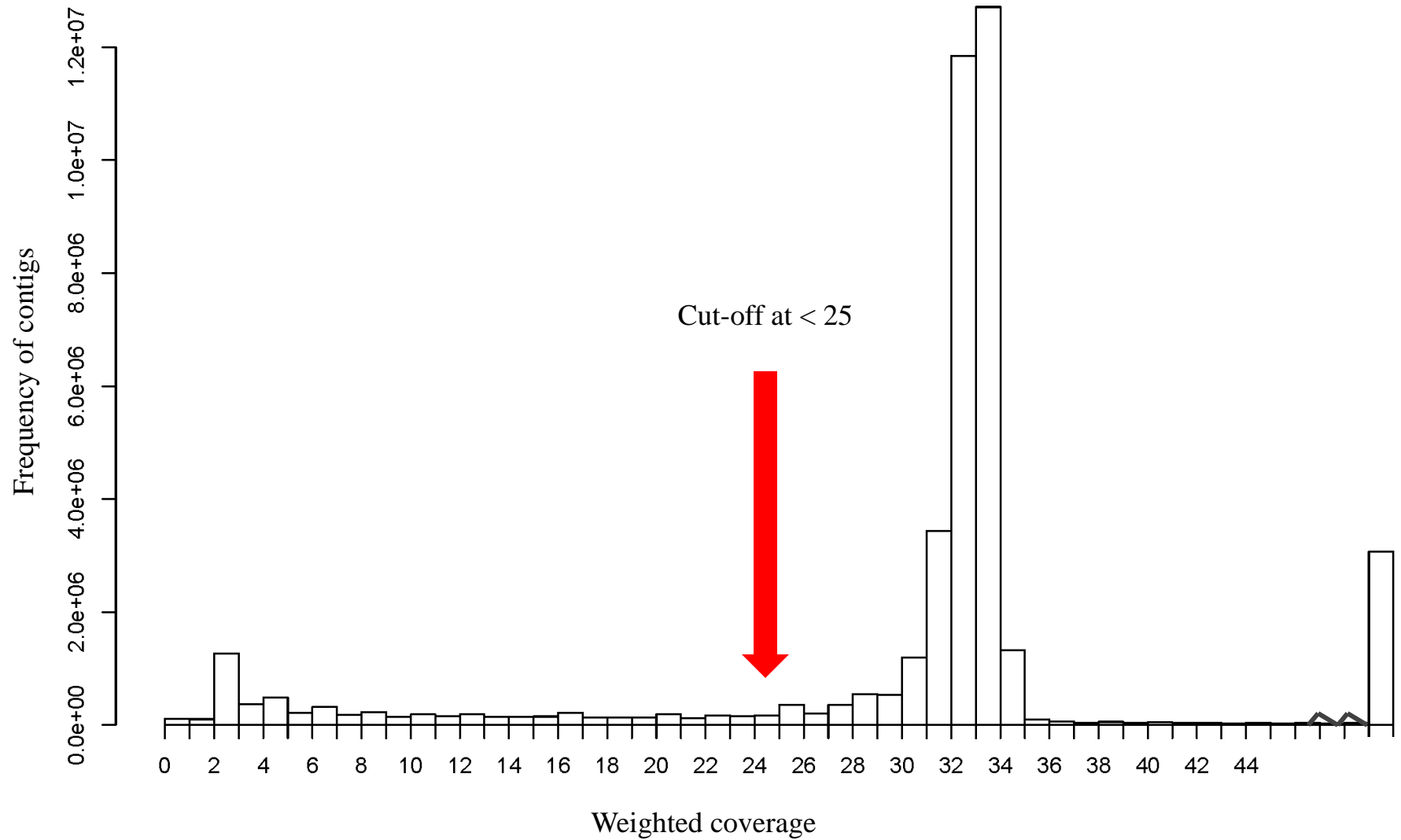

Supplement: Figure S3 — Average coverage of de novo assembled S1 contigs. Distribution of Velvet contig coverage. (0.03 MB PDF) [file pgen.1001189.s004.pdf]
