# Supplementary material for: Whole-Genome and Chromosome Evolution Associated with Host Adaptation and Speciation of the Wheat Pathogen Mycosphaerella graminicola
Source: PLoS Genet. 2010 Dec 23;6(12):e1001189. doi: 10.1371/journal.pgen.1001189 (PMC3009667; doi:10.1371/journal.pgen.1001189)
Supplement: Figure S2 — Synonymous (a) and intergenic (b) substitution rates across aligned chromosomes. (0.11 MB PDF) [file pgen.1001189.s003.pdf]

Figure S2a

Intergenic divergence

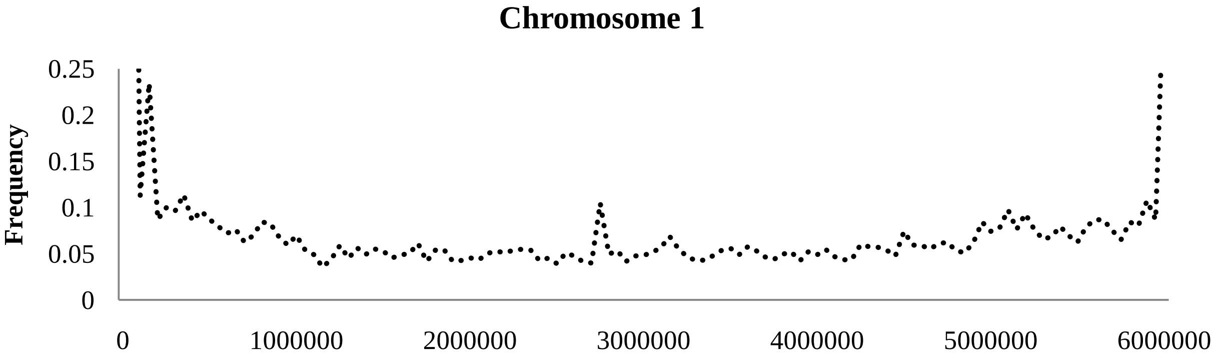


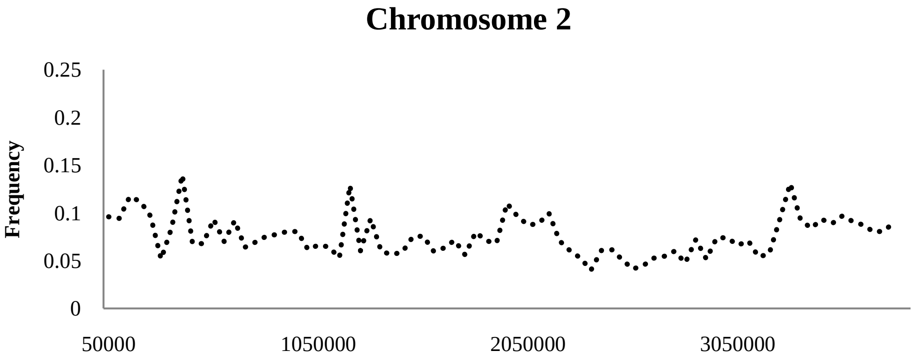


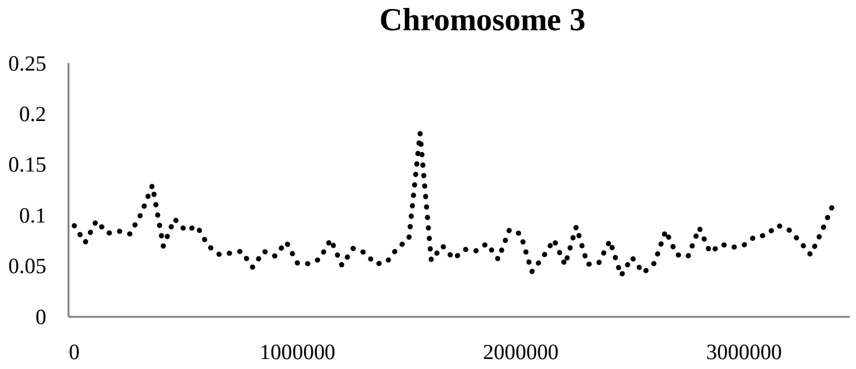


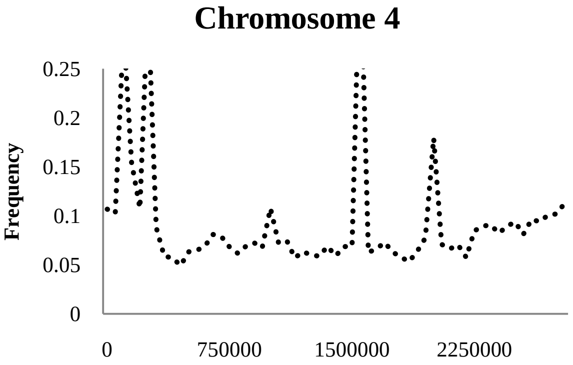


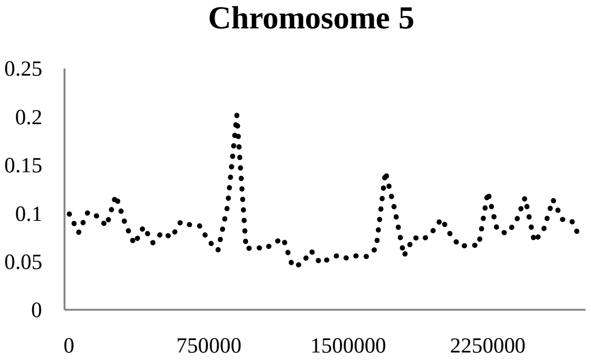


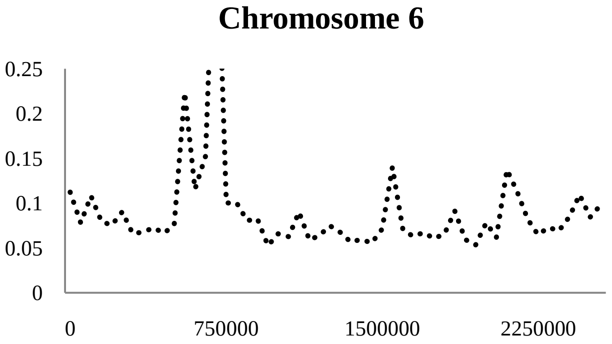

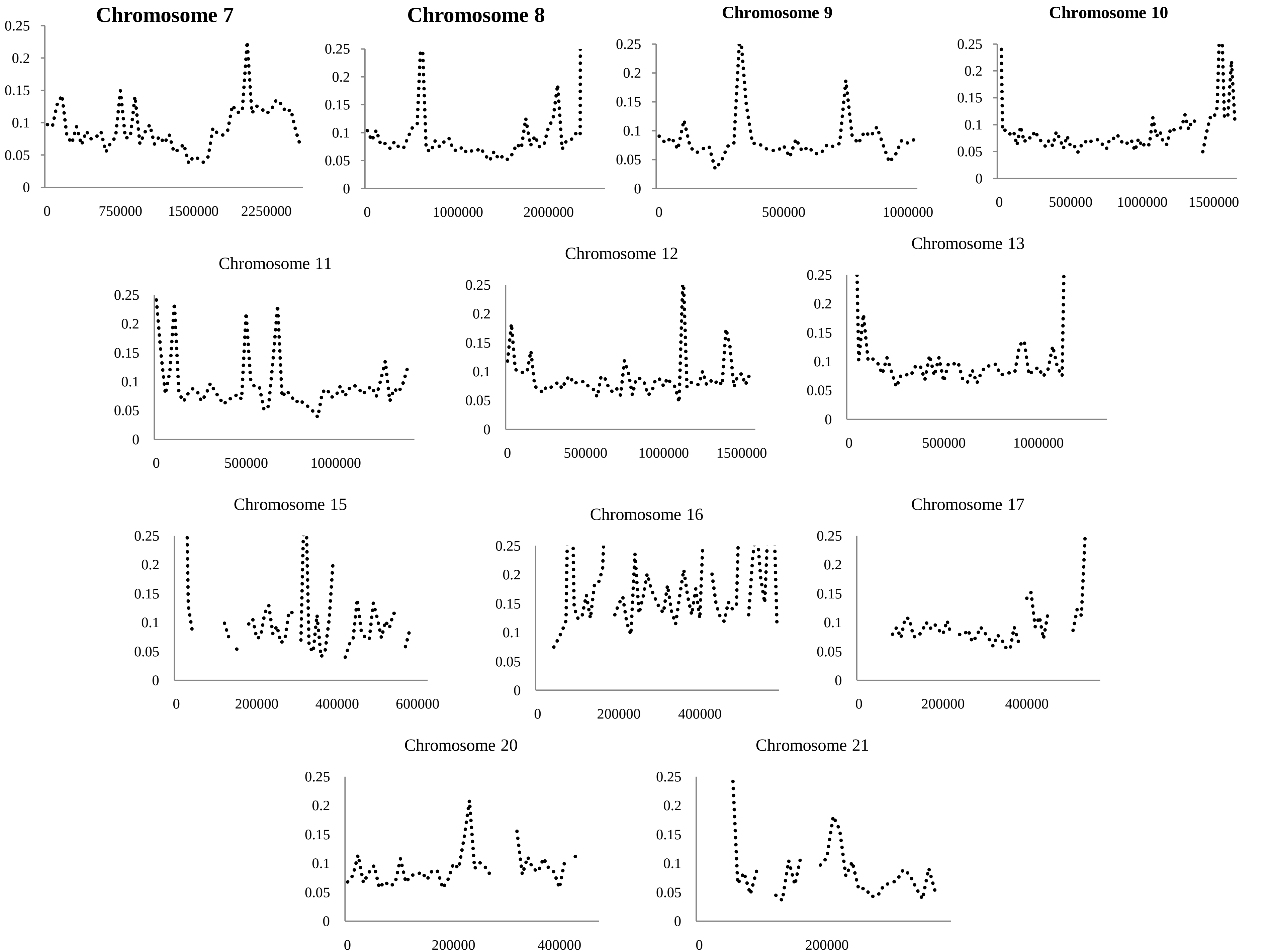
Chromosome 7
0 0.05 0.1 0.15 0.2 0.25
0 750000 1500000 2250000
Chromosome 8
0 1000000 2000000
Chromosome 9
0 500000 1000000
Chromosome 10
0 500000 1000000 1500000
Chromosome 11
0 500000 1000000
Chromosome 12
0 500000 1000000 1500000
Chromosome 13
0 500000 1000000
Chromosome 15
0 200000 400000 600000
Chromosome 16
0 200000 400000
Chromosome 17
0 200000 400000
Chromosome 20
0 200000 400000
Chromosome 21
0 200000

Figure S2b

Ks across chromosomes

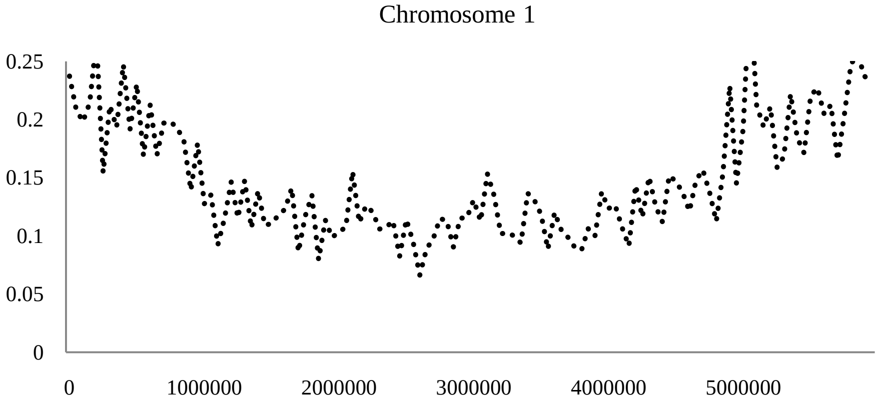


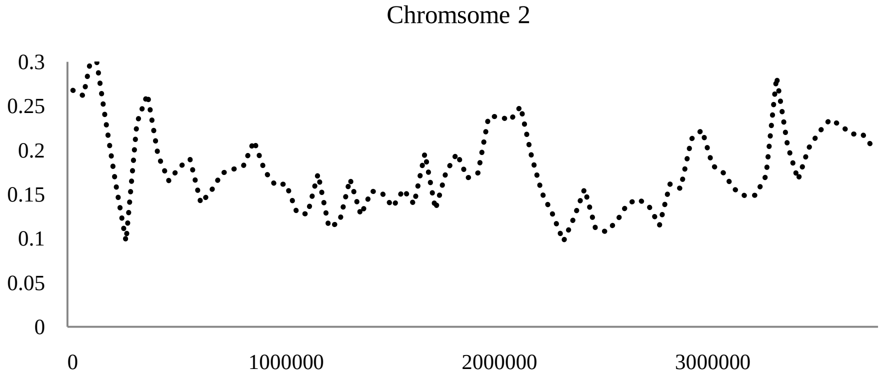


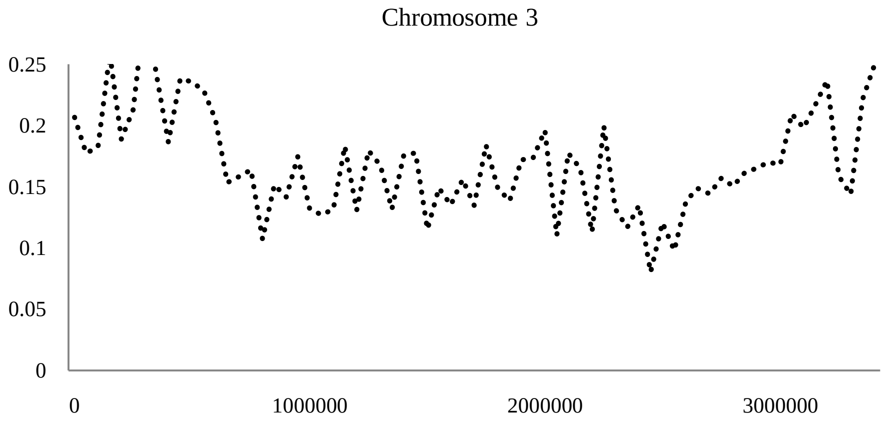


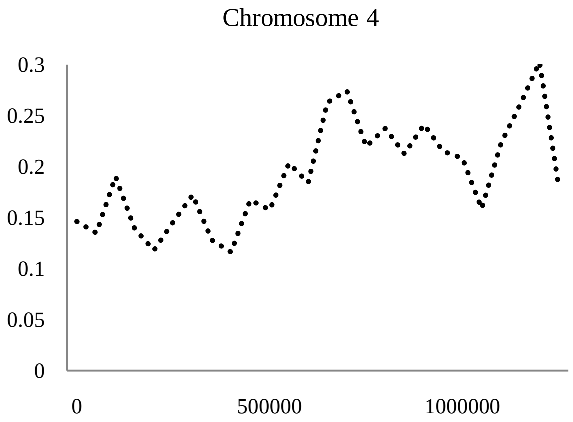


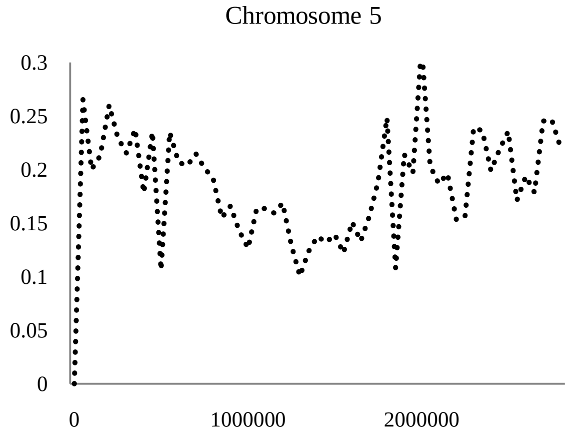


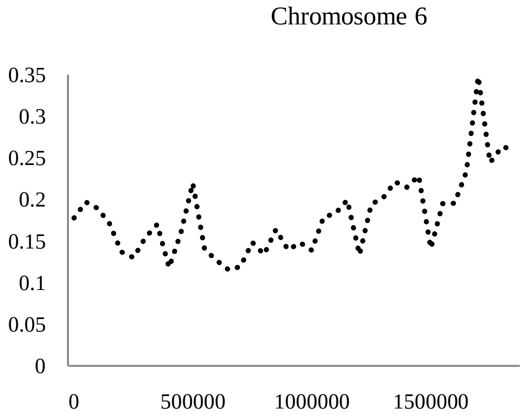

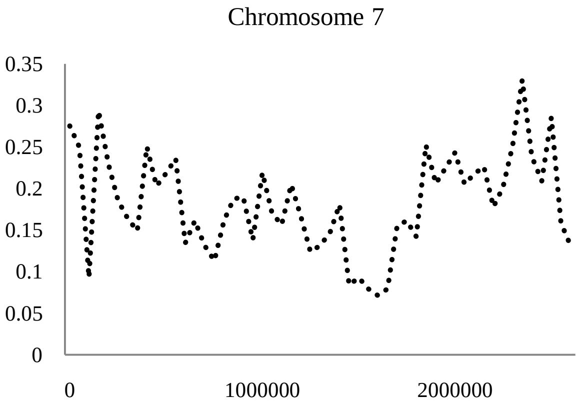
Chromosome 7
0.35
0.3
0.25
0.2
0.15
0.1
0.05
0
0
1000000
2000000

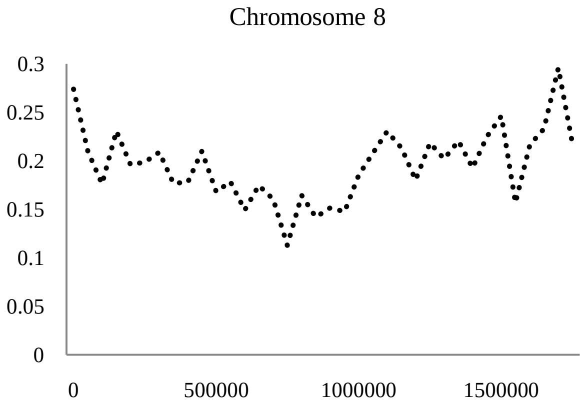
Chromosome 8
0.3
0.25
0.2
0.15
0.1
0.05
0
0
500000
1000000
1500000

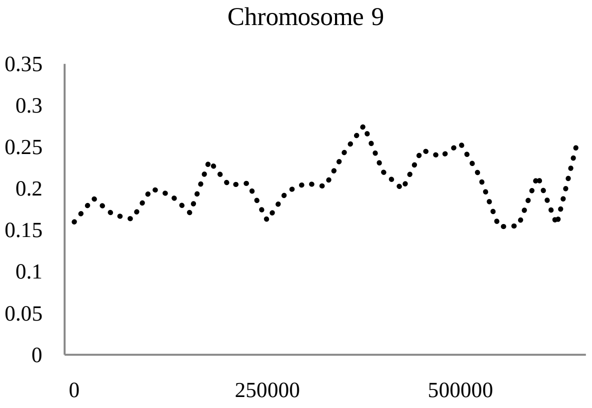
Chromosome 9
0.35
0.3
0.25
0.2
0.15
0.1
0.05
0
0
250000
500000

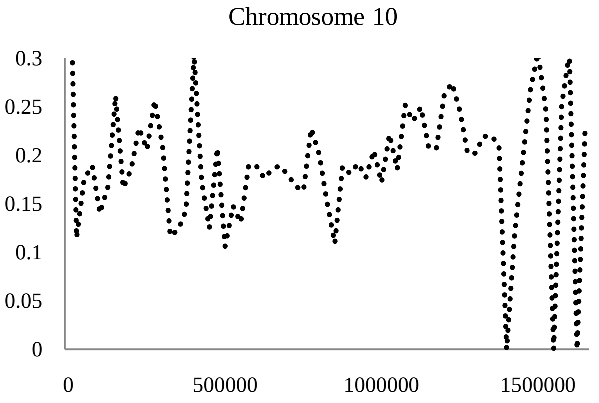
Chromosome 10
0.3
0.25
0.2
0.15
0.1
0.05
0
0
500000
1000000
1500000

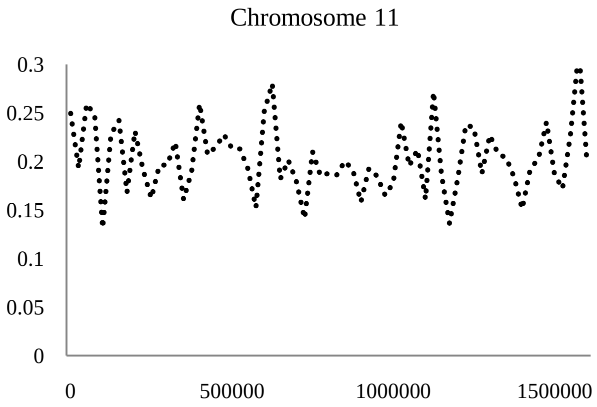
Chromosome 11
0.3
0.25
0.2
0.15
0.1
0.05
0
0
500000
1000000
1500000

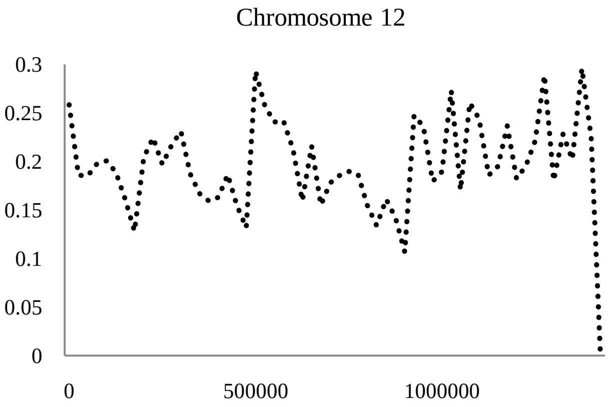
Chromosome 12
0.3
0.25
0.2
0.15
0.1
0.05
0
0
500000
1000000

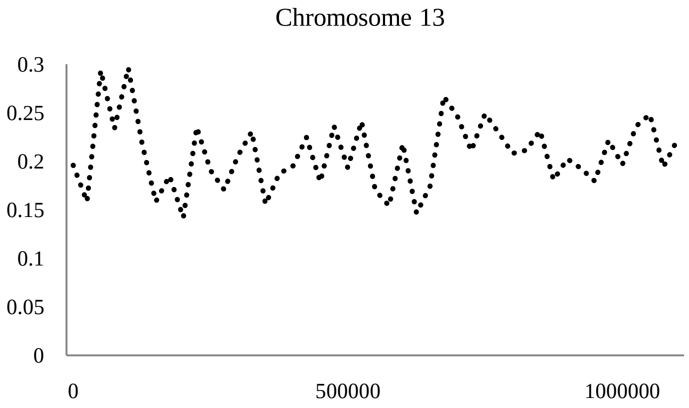
Chromosome 13
0.3
0.25
0.2
0.15
0.1
0.05
0
0
500000
1000000
