# Supplementary material for: Whole-Genome and Chromosome Evolution Associated with Host Adaptation and Speciation of the Wheat Pathogen Mycosphaerella graminicola
Source: PLoS Genet. 2010 Dec 23;6(12):e1001189. doi: 10.1371/journal.pgen.1001189 (PMC3009667; doi:10.1371/journal.pgen.1001189)
Supplement: Table S1 — Pathogenicity test of M. graminicola (STIR04-A26b) and S1 (STIR04-3.11.1) on wheat. The experiment was carried out with three repetitions where the percentage of leaf area covered by pycnidia was assessed. (0.01 MB PDF) [file pgen.1001189.s005.pdf]

Isolate	Experiment 1	Experiment 2	Experiment 3	Average %
STIR04-A26b	50%	50%	50%	50%
STIR04-3.11.1	1%	10%	1%	4%
