# Supplementary material for: Whole-Genome and Chromosome Evolution Associated with Host Adaptation and Speciation of the Wheat Pathogen Mycosphaerella graminicola
Source: PLoS Genet. 2010 Dec 23;6(12):e1001189. doi: 10.1371/journal.pgen.1001189 (PMC3009667; doi:10.1371/journal.pgen.1001189)
Supplement: Table S2 — Measures of synteny by counts of S1 contigs that align to one chromosome, two chromosomes, or three or more chromosomes. Counts are further divided into contigs aligning to essential chromosomes, dispensable or both types of chromosomes. (0.05 MB PDF) [file pgen.1001189.s006.pdf]

| Syntenty | Ranges of contig lenghts | | | |
|---|---|---|---|---|
| 1 chromosome hit | 1000-9999bp | 10000-49999bp | 50000-99999bp | ≥ 100000bp |
| Total | 203 | 269 | 122 | 68 |
| E[1)] | 165 | 238 | 116 | 68 |
| D[2)] | 38 | 31 | 6 | 0 |
| 2 chromosome hit | | | | |
| Total | 7 | 16 | 9 | 11 |
| EE | 3 | 7 | 8 | 11 |
| ED | 3 | 1 | 0 | 0 |
| DD | 1 | 8 | 1 | 0 |
| ≥ 3 chromosome hit | | | | |
| Total | 6 | 6 | 3 | 2 |
| EEE | 1 | 3 | 2 | 2 |
| EED | 4 | 1 | 0 | 0 |
| EDD | 0 | 1 | 0 | 0 |
| DDD | 1 | 1 | 1 | 0 |

[1)] Essential chromosome

[2)] Dispensable chromosome
