# Supplementary material for: Whole-Genome and Chromosome Evolution Associated with Host Adaptation and Speciation of the Wheat Pathogen Mycosphaerella graminicola
Source: PLoS Genet. 2010 Dec 23;6(12):e1001189. doi: 10.1371/journal.pgen.1001189 (PMC3009667; doi:10.1371/journal.pgen.1001189)
Supplement: Table S3 — The fraction of repetitive and non-repetitive DNA that is aligned in the S1-M. graminicola genome alignment. (0.01 MB PDF) [file pgen.1001189.s007.pdf]

	Repetitive		Non-repetitive	
	bp	freq	bp	freq
M. graminicola total	7123465	0.18	32728746	0.82
M. graminicola aligned	271101	0.01	27777342	0.70
S1 total	1409860	0.04	33836640	0.96
S1 aligned	271101	0.01	27777342	0.79
