# Supplementary material for: Whole-Genome and Chromosome Evolution Associated with Host Adaptation and Speciation of the Wheat Pathogen Mycosphaerella graminicola
Source: PLoS Genet. 2010 Dec 23;6(12):e1001189. doi: 10.1371/journal.pgen.1001189 (PMC3009667; doi:10.1371/journal.pgen.1001189)
Supplement: Table S4 — GO classification of annotated genes in the Mycosphaerella graminicola-S1 alignment (EuKaryotic Orthologous Groups http://genome.jgi-psf.org/Mycgr3/Mycgr3.home.html). (0.03 MB PDF) [file pgen.1001189.s008.pdf]

KOG class	Total annotated genes	Total aligned genes	Total non-aligned genes	Total codons	Average Ka/Ks	Average Ka	Average Ks
Extracellular structures	63	9	54	3396	0.205	0.032	0.161
Nuclear structure	112	109	3	69401	0.193	0.025	0.156
Defense mechanisms	118	117	1	50744	0.176	0.026	0.172
Chromatin structure and dynamics	145	98	47	50872	0.144	0.02	0.157
Cytoskeleton	206	158	48	92581	0.138	0.019	0.165
RNA processing and modification	332	314	18	188840	0.119	0.017	0.169
Function unknown	373	373	0	164811	0.115	0.017	0.172
Transcription	378	370	8	187213	0.11	0.015	0.153
Secondary metabolites biosynthesis, transport and catabolism	292	241	51	124578	0.109	0.019	0.193
Posttranslational modification, protein turnover, chaperones	628	557	71	236682	0.105	0.017	0.176
Signal transduction mechanisms	541	460	81	247711	0.104	0.016	0.166
Coenzyme transport and metabolism	111	77	34	30223	0.103	0.017	0.188
General function prediction only	1134	958	176	453682	0.097	0.015	0.176
Cell cycle control, cell division, chromosome partitioning	193	149	44	81502	0.095	0.013	0.153
Carbohydrate transport and metabolism	395	323	72	160916	0.093	0.016	0.194
Cell wall/membrane/envelope biogenesis	84	79	5	43848	0.088	0.013	0.175
Inorganic ion transport and metabolism	199	135	64	80174	0.086	0.014	0.184
Replication, recombination and repair	211	178	33	121153	0.085	0.014	0.184
Translation, ribosomal structure and biogenesis	351	350	1	144332	0.083	0.012	0.157
Nucleotide transport and metabolism	89	86	3	37814	0.08	0.015	0.197
Lipid transport and metabolism	345	305	40	165797	0.077	0.012	0.187
Amino acid transport and metabolism	320	275	45	132071	0.068	0.012	0.187
Cell motility	5	5	0	2814	0.068	0.01	0.162
Intracellular trafficking, secretion, and vesicular transport	316	250	66	128718	0.066	0.009	0.156
Energy production and conversion	334	327	7	128155	0.065	0.012	0.189
