# Supplementary material for: Whole-Genome and Chromosome Evolution Associated with Host Adaptation and Speciation of the Wheat Pathogen Mycosphaerella graminicola
Source: PLoS Genet. 2010 Dec 23;6(12):e1001189. doi: 10.1371/journal.pgen.1001189 (PMC3009667; doi:10.1371/journal.pgen.1001189)
Supplement: Table S5 — The 43 genes identified to have significant Ka > Ks in the M. graminicola and S1 alignment. (0.07 MB PDF) [file pgen.1001189.s009.pdf]

| Chromosome | Protein ID | Start position | End position | Nº of aligned codons | Nº of codons in predicted protein | Nº exons | EST support in *M. graminicola* | Nonsyn. mutations | Syn. mutations | Nei-Gojobori Ka/Ks[1)] | Nei-Gojobori Ka | Nei-Gojobori Ks | Nei-Gojobori Z-test |
|---|---|---|---|---|---|---|---|---|---|---|---|---|---|
| 1 | 88648 | 345834 | 347144 | 178 | 178 | 2 | - | 29 | 1 | 8.4660 | 0.0701 | 0.0083 | 4.1163 |
| 5 | 80707 | 657996 | 658613 | 125 | 125 | 1 | + | 24 | 1 | 6.9278 | 0.0825 | 0.0119 | 3.5281 |
| 9 | 105739 | 1229120 | 1229686 | 106 | 106 | 1 | + | 29 | 2 | 4.5537 | 0.1198 | 0.0263 | 3.3637 |
| 8 | 105480 | 1228554 | 1229995 | 77 | 78 | 1 | + | 14 | 1 | 4.5507 | 0.0803 | 0.0176 | 2.3198 |
| 8 | 105585 | 1882466 | 1882964 | 67 | 67 | 1 | + | 8 | 0 | 4.5357 | 0.0536 | 0.0118 | 1.7116 |
| 14 | 106599 | 671189 | 671736 | 106 | 119 | 1 | + | 19 | 1 | 3.9890 | 0.0801 | 0.0201 | 2.5227 |
| 17 | 97831 | 366251 | 366577 | 108 | 109 | 1 | - | 12 | 1 | 3.7622 | 0.0486 | 0.0129 | 1.9019 |
| 7 | 105176 | 983411 | 984541 | 140 | 140 | 1 | + | 11 | 1 | 3.6357 | 0.0348 | 0.0096 | 1.7974 |
| 18 | 97878 | 208583 | 209645 | 121 | 255 | 3 | - | 13 | 1 | 3.5967 | 0.0457 | 0.0127 | 1.8658 |
| 4 | 104171 | 1509984 | 1510593 | 53 | 53 | 1 | + | 19 | 1 | 3.5180 | 0.1585 | 0.0450 | 2.3882 |
| 6 | 93764 | 887981 | 888401 | 118 | 121 | 2 | - | 11 | 1 | 3.4401 | 0.0408 | 0.0119 | 1.7168 |
| 9 | 105694 | 727490 | 728225 | 159 | 161 | 2 | + | 18 | 2 | 3.1043 | 0.0508 | 0.0163 | 2.1043 |
| 1 | 33513 | 5083051 | 5083254 | 66 | 68 | 1 | + | 16 | 2 | 2.9746 | 0.1109 | 0.0373 | 2.0017 |
| 1 | 90306 | 5988847 | 5989320 | 88 | 158 | 1 | + | 22 | 2 | 2.8947 | 0.1053 | 0.0364 | 2.0891 |
| 6 | 104710 | 179204 | 179771 | 76 | 88 | 2 | + | 27 | 3 | 2.7990 | 0.1574 | 0.0563 | 2.4156 |
| 5 | 104488 | 1196254 | 1197312 | 155 | 156 | 2 | + | 25 | 3 | 2.7381 | 0.0714 | 0.0261 | 2.2381 |
| 20 | 98031 | 173524 | 175257 | 139 | 259 | 5 | - | 27 | 3 | 2.5223 | 0.0871 | 0.0345 | 2.1813 |
| 2 | 103264 | 1992714 | 1993521 | 109 | 111 | 2 | + | 37 | 4 | 2.5060 | 0.1499 | 0.0598 | 2.5660 |
| 20 | 98060 | 362354 | 362878 | 132 | 152 | 2 | - | 23 | 3 | 2.4759 | 0.0768 | 0.0310 | 1.9565 |
| 7 | 100838[2)] | 1785582 | 1786221 | 115 | 115 | 2 | + | 21 | 3 | 2.4706 | 0.0824 | 0.0333 | 1.9163 |
| 1 | 90212 | 5612533 | 5613441 | 82 | 120 | 2 | + | 25 | 3 | 2.3849 | 0.1376 | 0.0577 | 2.0384 |
| 21 | 98101 | 253102 | 253948 | 70 | 120 | 2 | - | 19 | 2 | 2.3111 | 0.1204 | 0.0521 | 1.6650 |
| 9 | 105817 | 2092225 | 2092890 | 87 | 120 | 1 | + | 19 | 3 | 2.2047 | 0.1043 | 0.0473 | 1.7115 |
| 17 | 97821 | 302722 | 303180 | 116 | 119 | 2 | - | 20 | 3 | 2.1770 | 0.0762 | 0.0350 | 1.7010 |
| 1 | 83337 | 534698 | 534961 | 88 | 88 | 1 | + | 18 | 3 | 2.0135 | 0.0911 | 0.0452 | 1.4015 |
| 12 | 106257 | 176773 | 177370 | 96 | 98 | 1 | + | 28 | 5 | 1.7696 | 0.1323 | 0.0748 | 1.6746 |
| 20 | 98059 | 360675 | 361856 | 200 | 394 | 1 | - | 55 | 10 | 1.6948 | 0.1199 | 0.0708 | 1.8643 |
| 20 | 98027 | 152654 | 153616 | 293 | 321 | 1 | - | 45 | 7 | 1.6931 | 0.0663 | 0.0392 | 1.7099 |
| 1 | 88614 | 211916 | 212437 | 95 | 174 | 1 | - | 33 | 6 | 1.6150 | 0.1544 | 0.0956 | 1.3584 |
| 11 | 96691 | 938961 | 939374 | 138 | 138 | 1 | - | 55 | 10 | 1.6075 | 0.1753 | 0.1091 | 1.7475 |
| 14 | 106598 | 668801 | 670705 | 440 | 445 | 3 | + | 84 | 16 | 1.6044 | 0.0842 | 0.0525 | 2.0817 |
| 1 | 102326 | 2430482 | 2430800 | 71 | 74 | 1 | + | 3 | 0 | - | 0.0176 | 0.0000 | 1.7475 |
| 1 | 102715 | 4102944 | 4103468 | 59 | 62 | 1 | + | 3 | 0 | - | 0.0227 | 0.0000 | 1.7521 |
| 1 | 90122 | 5331411 | 5332291 | 107 | 108 | 3 | - | 4 | 0 | - | 0.0161 | 0.0000 | 2.0163 |
| 2 | 108330 | 3382976 | 3384058 | 40 | 64 | 3 | - | 10 | 0 | - | 0.1075 | 0.0000 | 3.3474 |
| 3 | 79746 | 55468 | 55976 | 47 | 76 | 2 | + | 3 | 0 | - | 0.0285 | 0.0000 | 1.7573 |
| 3 | 103617 | 907856 | 908908 | 92 | 92 | 1 | + | 8 | 0 | - | 0.0380 | 0.0000 | 2.8837 |
| 4 | 104217 | 1865219 | 1865677 | 76 | 76 | 1 | + | 9 | 0 | - | 0.0520 | 0.0000 | 3.0812 |
| 9 | 82188 | 1590928 | 1591453 | 52 | 52 | 1 | + | 16 | 0 | - | 0.1341 | 0.0000 | 4.2985 |
| 9 | 82188 | 1590928 | 1591453 | 49 | 52 | 1 | + | 4 | 0 | - | 0.0353 | 0.0000 | 2.0363 |
| 11 | 106231 | 1469937 | 1470567 | 75 | 75 | 1 | + | 3 | 0 | - | 0.0171 | 0.0000 | 1.7471 |
| 12 | 106324 | 564439 | 564970 | 57 | 57 | 1 | + | 10 | 0 | - | 0.0759 | 0.0000 | 3.2897 |
| 20 | 98001 | 35726 | 36951 | 13 | 254 | 4 | - | 4 | 0 | - | 0.1290 | 0.0000 | 2.1430 |

[1)] Not calculated for synonymous muations = 0

[2)] Signal peptide
