# Supplementary material for: Whole-Genome and Chromosome Evolution Associated with Host Adaptation and Speciation of the Wheat Pathogen Mycosphaerella graminicola
Source: PLoS Genet. 2010 Dec 23;6(12):e1001189. doi: 10.1371/journal.pgen.1001189 (PMC3009667; doi:10.1371/journal.pgen.1001189)
Supplement: Table S6 — Detailed sequencing and analysis of the MAT1-1 locus. (0.01 MB PDF) [file pgen.1001189.s010.pdf]

	No of sequences *M. graminicola* / S1	Nucleotides	No of fixed differences	Polymorphic sites in *M. graminicola*	Polymorphic sites in S1	Synonymous substitutions	Nonsynonymous substitutions
Mating type 1-1 gene	1 / 1	888	12			7	5
Mating type locus 7Kb	1 / 1	7540	423				
Partial mating type 1-1 gene	8 / 9	511	9	2	0	6	3
MT 1 R4F3	116 / 21	494	10	3	0		
MT2 R3F2	76 / 22	511	13	1	0		
